# Too complicated for the field? Measuring quality of care in humanitarian aid settings

**DOI:** 10.3402/gha.v6i0.20311

**Published:** 2013-05-16

**Authors:** Roland Kersten, Götz Bosse, Frank Dörner, Andrej Slavuckij, Gustavo Fernandez, Michael Marx

**Affiliations:** 1 Independent International Health Consultant, Berlin, Germany; 2 Department of Anaesthesiology and Intensive Care Medicine, Charité-Universitätsmedizin Berlin, Germany; 3 Médecins Sans Frontières, Berlin, Germany; 4 Médecins Sans Frontières, Geneva, Switzerland; 5 Institute of Public Health, University of Heidelberg, Heidelberg, Germany

**Keywords:** humanitarian, quality of care, evaluation, developing countries, low-income, health care, health, assessment, quality, process

## Abstract

While quality of care is a major concern in the western world, not many studies investigate this topic in low-income countries. Even less is known about the quality of care in humanitarian aid settings, where additional challenges from natural or manmade disasters contribute to additional challenges. This study tried to address this gap by introducing a new approach to systematically measure quality of care in a project of Médecins Sans Frontières (MSF) in Agok area, between South Sudan and Sudan. Our objective was to obtain a valid snapshot of quality of care for a MSF project in three weeks that has the potential to serve as a baseline for quality improvement strategies. The evaluation followed a cross-sectional study design to assess structural, process and outcome quality according to Donabedian's criteria of quality of care. A bundle of well-established methods for collection of quantitative and qualitative data was used to assess the project by following a triangulated mixed-methods approach. Mean structural quality scored 73% of expected performance level and mean process quality 59%. The overall mortality rate for the hospital was 3.6%. On average, less complicated cases got a better level of care than patients who were seriously ill. Significant motivational issues were discovered in staff interviews potentially affecting quality of care. The tool appeared to be quick, feasible and effective in judging quality of care in the selected project. To tap the whole potential of the approach a re-evaluation should be carried out to assess the effectiveness of implemented improvement strategies in Agok. To confirm the usefulness of the approach, more studies are needed covering the variety of different humanitarian aid settings.

Quality of care seems to be not only difficult to define but also not easy to measure. In the Western world, the assessment of quality in health care has nevertheless become a routine. To a lesser extent, quality of care is measured in developing countries also, but when it comes to humanitarian aid settings, difficulties deriving from disaster settings seem to hinder effective evaluations of quality of care (1).

Many non-governmental organisations around the world have realised the urgent need for measuring and improving quality of care in humanitarian aid projects; Médecins Sans Frontières (MSF), which provides emergency medical assistance to populations in danger in more than 60 countries, is one of them. Because MSF is constantly making efforts to improve quality of care in their projects, they agreed to conduct a pilot study in one of their programmes to demonstrate that quality of care can be measured effectively and comprehensively with this new approach in the context of humanitarian aid. The project chosen was situated in Agok, Abyei area, a disputed zone between the Republic of the Sudan and the Republic of South Sudan.

## Context from the literature

The issue of low performance in health care in developing countries is widespread, and strategies for improvement face complex difficulties on implementation (2). While evaluation of quality of care is a major concern in the Western world, not many studies have been carried out in developing countries and their validity is varying (3). Leatherman claims that while ‘modern approaches to improving quality are increasingly used globally, their appropriateness for resource poor settings has received little attention and their adoption remains sporadic’ (4).

Proceeding from the situation in developing countries to humanitarian aid contexts, even less is known about quality of care. This is not to say that quality of care is not evaluated in those settings. Crisp (5) even argues that ‘humanitarian evaluations have now become big business’, but their effectiveness, especially for improving processes in patient care remain questionable and only a few approaches are published. What are the reasons for this lack of knowledge on quality of care in humanitarian aid settings? Banatvala argues that due to the many other priorities in emergency settings, evaluation of quality of care has long been neglected in this particular field of health care. Obviously, factors deriving from natural or man-made disasters contribute to additional challenges in those situations (1) and more recent literature is also confirming ‘In disaster settings, insuring quality of care is extremely challenging’ (6).

Unfortunately, it seems to be crucial, especially in humanitarian aid situations, to be able to come to sound decisions as fast as possible. To be forced to decide in spite of a severe lack of data is part of the daily frustration of health care workers in emergency settings (7). However, especially in this difficult environment, action cannot be delayed. This sometimes leads to mistakes, often in the initial phase of a project, which can be difficult to correct later.

One way to make sure that patients get the care they need and the most effective treatment possible in the given context is the introduction and use of clinical guidelines, which are seen in the literature as an important link between scientific evidence and clinical practice (8). It has been demonstrated that standards and protocols lead to improved quality of care; in 55 out of 59 guideline studies, Grimshaw & Russel identified at least one beneficial change for health care provision (9). For this reason, evaluation of adherence to guidelines became a major methodological feature in this study, taking the degree to what guidelines are implemented and followed as one measure for quality of care. Simply having guidelines developed and making them available does not guarantee their use (10).

## Local context

Today, many observers see health care in South Sudan as being at crossroads (11). After a period in 2012 with considerable low levels of violence in Abyei Administrative Area, tension was rising again after the decision of the government in South Sudan to turn off the oil production and with it 98% of the national revenue. This happened following pricing disputes with the northern neighbour, who owns the refineries and pipelines. The achievements in development that have been accomplished with the aid of the international community are again seriously in danger.

In Agok, like in other parts of (South) Sudan, only a low percentage of the population has access to health care. According to country reports of MSF, which has been working in Sudan since 1983, there is a lack of access to safe drinking water, especially during the rainy season, and access to and use of latrines is low. The public health services are facing chronic malfunction with low coverage in terms of primary and secondary health care, lack of skilled human resources as well as shortages of drugs, vaccines, and material supplies. The level of care at public health structures is basic at best and hygiene standards are very low.

Since August 2008, the medical programme of MSF-Switzerland in Agok offers primary and secondary health care, fixed outreach site, and mobile clinics as well as emergency interventions to an estimated population of 100,000 people. Services include in- and outpatient departments, comprising mother and child health with ante- and post-natal care, vaccinations, nutritional support, communicable disease intervention, community health care, trauma, surgical and referral services. Specific health needs such as tuberculosis (TB) and outbreak responses are also addressed. In 2012, 91 beds were provided in Agok hospital – 26 beds in the Inpatient Department (IPD), 36 beds in the Inpatient Therapeutic Feeding Centre (ITFC), 15 beds in Maternity, 8 beds in Surgical/Recovery, and 6 beds in TB Department.

## Methodology

**Fig. 1:**
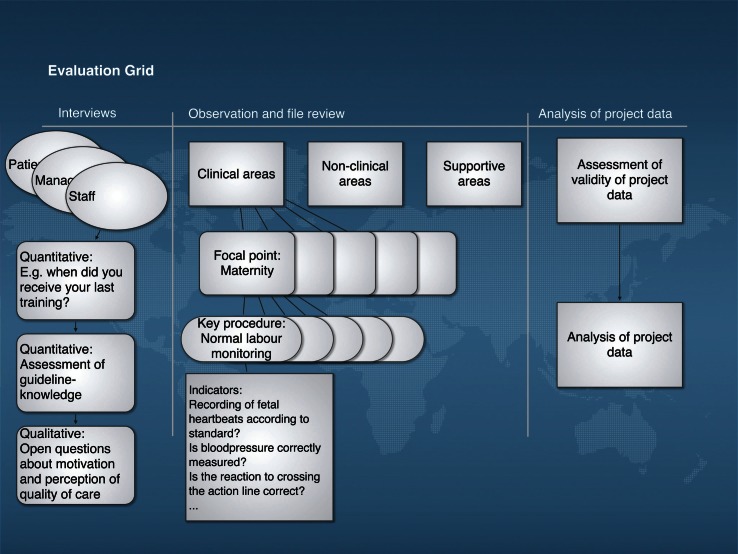
Evaluation grid with indicator examples.

The method originates from a hospital assessment tool published by Bosse et al. in 2011 (12). The evaluation followed a cross-sectional study design with some retrospective aspects. According to the set-up of the Agok project, 11 focal points were identified to provide a comprehensive picture of quality of care in the project. They were divided into clinical, non-clinical, and supportive areas. Several key procedures described each focal point. The focal point ‘maternity’, for example, comprised 12 key procedures, one of them being ‘monitoring normal deliveries with partograph’. Each key procedure was a composite variable made up of several indicators, for example, ‘was outcome of the baby documented on the partograph? ‘ (see supplemental file 1). Structural, and outcome quality of the project was evaluated according to Donabedian's criteria of quality of care (13) with a focus on process quality. Agok hospital, including outpatient department and mobile clinics, was assessed by one researcher in 11 working days. Sampling for data collection followed an opportunity sampling approach. Whenever a key procedure (e.g. a ward round) was coming up and the researcher was available, all related processes were systematically assessed. Whenever possible, key procedures were observed several times, but there were also processes that could only be assessed once during the evaluation period. A combination of several well-established methods were used to obtain a trustworthy snapshot of the situation in the project (Fig. 1): observations of health care encounters were recorded (14), using structured checklists in a non-participatory observational approach, measuring process quality. Structured inventory checklists were used to assess the structural quality of each department in the project, assessing completeness and functionality of equipment and supplies. Checklists were also used to assess 67 patient files, analysing clinical data of the past six months, and comparing this information to the MSF standard. Indicators used for all these checklists were based on 17 current MSF guidelines, one national South Sudanese Guideline, and one WHO guideline (see supplemental file 2). To measure the quality of health care performance, the catalogue of indicators served as the expected performance level (EPL) against which the actual performance level (APL) was being compared. For the purpose of quantitative analysis, each indicator was labelled with a value from 0 to 2 points, where 0 points meant not performed according to standard, 1 point meant not fully performed according to standard, and 2 points stood for performance according to the MSF standard. Each performance, including availability, was graded on these values by comparing the actual performance to the agreed-upon standard from guidelines. Outcome data of the project was assembled from routine MSF databases and reports. Collection and processing of data was observed to estimate the soundness of the available outcome data.

Semi-structured interviews completed data collection for the quantitative items. Three sets of interview guides were used and sampling for interviews also followed an opportunity sampling approach according to the availability of the researcher, suitable candidates, and the translator. Four interviews were conducted with project managers on different levels, 9 with health care workers, and 10 with patients after they were discharged. For qualitative analysis, open-ended interview questions were designed to find out reasons for the quantitative findings as well as levels of motivation and satisfaction. In the quantitative part of the staff interviews, knowledge according to MSF treatment guidelines was assessed using simple questions directly related to the daily work of the individual care provider. All interview candidates were carefully informed and patients signed a written consent before participating in the interview. Participants were both men and women, all older than 21 years and all contractually capable. For all patients and some staff who did not speak English, interviews were translated from Arabic or Dinka into English and back. For validation, they were later compared with the translation of an independent translator. Interviews were conducted in English (see supplemental file 3)

### Analysis of data

For analysis of all checklists, quantitative results were expressed as numbers and percentage giving all items the same power. Weighting of indicators was achieved by repetition of certain items in the evaluation process. For each item, a score from 0 to 2 was assessed. These scores were counted and represented the actual performance level that was compared to the highest achievable score, the EPL. Likewise, checklists from the review of clinical files were analysed to support findings from observations. Calculations and drawing of graphs for this article was performed using Microsoft^®^ Excel for Mac, 2011and Grafio, Ten Touch Ltd.

Interviews were analysed quantitatively and qualitatively. In the case of identical answers, frequency was indicated as a percentage of all answers. Similar answers were grouped into themes and patterns and possible interpretations were collected. Single citations illustrated the themes. Health workers’ knowledge of MSF guidelines was assessed by comparing the completeness of their description to the information provided by MSF guidelines. This was also expressed as a percentage.

### Limitations

A possible bias for the methods chosen could have been the presence of an external observer in the project. This is a well-known bias in observational studies, also called the observer's paradox (15). However, it is considered to have only limited effects, because of the long time of observation. In addition, the observer was part of MSF during the field trip and a medical expert merging as much as possible into the setting. Also, MSF staff are very much used to field visitors observing their daily work. To further address this potential bias, triangulation was used to collect data and enhance the soundness of the study.

The quality of routinely collected outcome data in the field could not be judged. Observations on documentation and data processing gave the impression that cogency of available data was limited. Relevant gaps also occurred in the continuously collected programme data, due to evacuations and interruptions of services in the past related to impaired security. These facts limited the interpretability of project outcome data for this evaluation.

### Ethical considerations

Due to the unsolved political situation in the Abyei area, no relevant authorities were in place to apply for ethical clearance locally. As most parts of the evaluation is considered as internal assessment of a MSF programme, only the interviews with patients could raise ethical questions. To address this issue as carefully as possible, informed written and signed consent from all patients was obtained, all the while being aware of the special vulnerable population in humanitarian aid settings (16). Participants were fully informed about the purpose of the assessment, their anonymity, and their right to withdraw at any stage. The study was carried out in accordance with the Helsinki Declaration (17) (see supplemental file 4).

## Results

At the end of the assessment, supervisors in Agok and coordinators in Juba were confronted with raw data highlighting the strength and weaknesses of the project and making suggestions for possible improvement strategies. Performance from each department and patients’ perceptions were presented, guarding the anonymity of the interviewed individual. Management in the Agok project, coordinators in Juba, and those at the MSF-Switzerland Headquarters in Geneva were provided with a detailed report shortly after the evaluation of the project. This report provided a comprehensive snapshot of all measured aspects related to quality of care in the project and made several detailed suggestions for improvement. Only some selected results are shown here as an example; percentages of performance levels combine the results from observations and file review presented as means and corresponding standard deviations. *n* corresponds to the number of different key procedures for process quality results; how many times a key procedure could have been observed varied from 1 to 5. *n* for structural quality results present the number of key items checked.

**Fig. 2:**
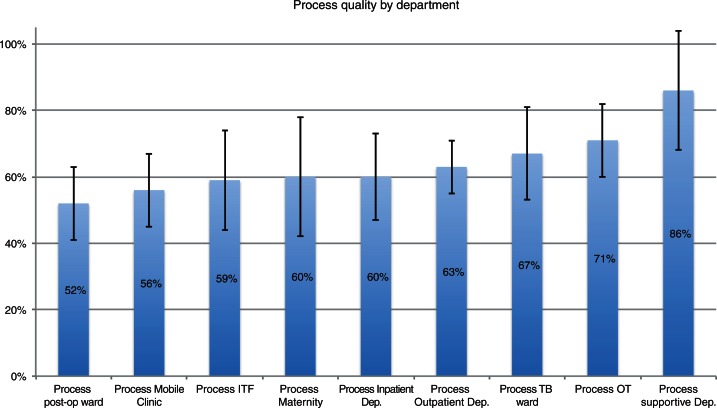
Results of process quality assessment for each department. Measured performance level compared to performance demanded in guidelines in % and standard deviations.

The observational checklists for structural and process quality assessment, as well as the checklists for patient file review provided detailed quantitative results. Mean structural quality for all assessed areas of the project scored 73% (*n*=273, SD±7%) of EPL, and mean process quality reached 59% (*n*=324, SD±9%). The overall mortality rate for the hospital was 3.6% in 2011. Structural quality was impaired by the storage condition of drugs and equipment on wards, missing supplies in some departments, and poor cleaning of low risk areas like ‘normal’ wards. Process quality for supportive departments (laboratory and pharmacy) scored 86% (*n*=7, SD±18%) of EPL while clinical areas showed more deficiencies (Fig. 2). For specialised areas like the TB ward (67%, *n*=14, SD±14%) and the OT (71%, *n*=15, SD±11%), scores for process quality were better than for ‘normal’ wards like the Inpatient Department (60%, *n*=15, SD±13%).

The best process quality was assessed for supportive departments, such as the laboratory (85%, *n*=7, SD±4%). On average, less complicated cases (e.g. uncomplicated malaria) got a better level of care 79% (23 cases, SD 8%) than patients who were seriously ill, e.g. in a coma (50%, 10 cases, SD 7%). General nursing care procedures scored 54% (*n*=12, SD±7%) and hygiene procedures marked 41% (*n*=13, SD±11%) of EPL. Specialised care like treatment of sick neonates, that was performed on a maternity ward, showed the most shortcomings with a score of 33% (*n*=4, SD±2%; ).

Interviews with health care workers revealed results that provided explanations for findings coming from the observational part. Theoretical knowledge was low, with eight out of nine health care workers scoring below 20% of expected guideline understanding. Nobody saw his or her own performance as key for better quality, but motivational problems were seen by 55% as impairing quality of care. As a reason for low motivation, health care workers mentioned high workload and low salary in 44% of interviews. The strongest motivator for all national staff was the fact that they were able to help their own people.

All interviewed managers were highly motivated and satisfied with their job. Strengths were seen in supportive departments and performance of expatriates, weaknesses were perceived in nursing skills, hygiene and in many cases performance of national staff, all in line with the observed results.

Patients were very satisfied with the service in the Agok project. Friendliness of staff and effectiveness of treatment was especially appreciated. The only complaint was about dirty latrines mentioned by 10% of patients. All patients felt well informed on discharge; nevertheless, just 70% of patients understood their diagnosis, while 60% knew in what cases to come back to the hospital, and only 40% understood how to administer their drugs at home.

## Discussion

### Structural quality

The structural quality in the Agok project scored better than process quality (73 vs. 59%), but still was below the expected standard. Departments that were no longer based in tents, as the construction of permanent buildings was on-going, obtained better results. This fuels the hope that when construction is finalised, structural quality will improve in the near future. A minimum level of structural quality is needed to provide a meaningful level of care (13). This essential level seems to be available in the Agok hospital but in certain areas, such as storage of drugs or equipment on wards, structural quality is in urgent need of improvement.

### Process quality

To measure a considerably low level of process quality did not come as a surprise. Perception on quality of care by key informants matched with these findings and results from a district hospital in Tanzania, evaluated with a parallel approach showed similar scores (12). In spite of obstacles such as instability, insecurity, and constant lack of resources, MSF seems to be able to provide a level of process quality in a humanitarian aid setting that can be compared to the stable context of an African district hospital in Tanzania.

Process quality was significantly influenced through the performance quality of the individual health worker. At the same time, in interviews, no member of staff made the link between his own performance and overall quality of care. The best level of care was observed for patients without risk factors and non-emergencies, such as patients who came because of uncomplicated malaria. Key procedures, which included continuous monitoring, the care for sick neonates, or postoperative care, showed a severe lack of continuous high-level performance. Unfortunately, it is these complicated and more severe cases that need the medical attention of a secondary health care facility like the Agok hospital.

If there is a general feeling in a project that quality of care might be low, it is in many cases structural quality that gets the most attention (18). This was the case in the Agok project also. Quality of care was perceived as being problematic and to do something about it, a lot of structural improvement was going to happen. As structural quality is seen as the basis for any other aspect of quality of care, this is good and this is necessary. However, even without construction being completed, structural quality scored significantly better than process quality. This does not mean that nothing was done to improve processes in the programme. Having a training nurse in the project was definitely a step in this direction. However, in order to improve quality of care in the Agok project effectively and in a sustainable way, one of the major recommendations was to shift the focus from structural to process quality. Even though that means addressing issues where there are no quick fixes and where financial investments are needed, like the low knowledge of national staff or motivational and attitudinal problems.

Adherence to guidelines was a major aspect for the measurement of process quality in this study. As the results suggest, adherence to guidelines had room for improvement in the Agok project. Among other reasons, clinical guidelines have been introduced to reduce errors in medicine, and it is well known that medical errors are not a problem of developing countries alone (19). One way to prevent or reduce the occurrence of errors are clinical guidelines, but their implementation ‘faces complex difficulties’ (20). Nevertheless, trying to understand people's motivation and difficulties to work according to guidelines remains inevitable.

In Agok, many different aspects impaired the performance level of quality of care. The perception that salaries were low compared to a workload that was perceived as being too heavy, combined with a low guideline-knowledge of health workers contributed to the measured results. The problem is known for its complexity: ‘measurable improvements in the quality of delivered care and reductions in medical errors have been variable and modest in most cases. Multiple barriers to the implementation of patient safety and error reduction initiatives have been identified in the literature’ (21). One way to overcome those difficulties seems to be performance feedback (22), which has been shown to have ‘a significant effect on compliance with standards’ (23). Research evidence has shown that clinical audits and feedback can improve clinical practice (10). This study was providing feedback and recommendations to the field. Whether this will have an effect on guideline adherence could only be measured with a re-evaluation in the future.

### Outcome quality

Outcome in terms of morbidity and mortality in the hospital could not be compared to local data, due to the collapse of public health services in the Abyei area. Generally, the usefulness of outcome data as quality indicators for humanitarian settings seems questionable and there is an on-going debate mainly because compounding factors are extremely difficult to control.

Nevertheless, especially for comparison over time, outcome quality was not neglected. Hospital outcome data was listed whenever available, but their interpretation remains difficult.

## Conclusion

The bundle of methods used in Agok appeared to be quick, feasible, and effective in judging quality of care in the selected project. After the assessment, a comprehensive picture of quality of care in the project could be presented, showing detailed strength and weaknesses. Reasons for the encountered situation could also be offered to explain some of the findings by showing knowledge, motivation, and attitude of health workers. Recommendations were presented, derived from measured priorities, showing ways for improvement whenever possible and appropriate.

Whether this way of evaluating humanitarian aid settings becomes an effective tool cannot be answered at this stage. At the moment, the results can serve as a baseline for improvement measures; they can help to prioritise problems and to allocate resources. To tap the whole potential of this approach, re-evaluations should be planned to assess the effectiveness of implemented improvement measures in Agok. Aiming for a long-term strategy to manage quality of care in Agok and other humanitarian aid projects, implementation of a quality improvement circle and training for self-evaluation with the tool should be aimed for.

As the set-up, scope, and context of humanitarian aid settings differ a lot, other situations need to be investigated to reach conclusive judgements about the effectiveness of this approach. However, the study could demonstrate that quality of care in humanitarian aid can be evaluated in a way that results can have the potential to help programme managers to implement improvement measures.

According to our experience, it does not seem to be too complicated to measure quality of care effectively in the field. Quite certainly, there are no valid reasons to exclude beneficiaries in humanitarian aid programmes from systematically evaluated health care any longer.
